# DDX3 enhances oncogenic KRAS-induced tumor invasion in colorectal cancer via the β-catenin/ZEB1 axis

**DOI:** 10.18632/oncotarget.8143

**Published:** 2016-03-17

**Authors:** De-Wei Wu, Po-Lin Lin, Ya-Wen Cheng, Chi-Chou Huang, Lee Wang, Huei Lee

**Affiliations:** ^1^ Graduate Institute of Cancer Biology and Drug Discovery, Taipei Medical University, Taipei, Taiwan; ^2^ Institute of Medicine, Chung Shan Medical University, Taichung, Taiwan; ^3^ School of Medicine, Chung Shan Medical University, Taichung, Taiwan; ^4^ Department of Surgery, Division of Colon and Rectum, Chung Shan Medical University Hospital, Taichung, Taiwan; ^5^ School of Public Health, Chung Shan Medical University, Taichung, Taiwan

**Keywords:** DDX3, KRAS, β-catenin, AKT, colorectal cancer

## Abstract

DDX3 plays a dual role in colorectal cancer; however, the role and underlying mechanism of DDX3 in colorectal tumorigenesis remains unclear. Here, we provide evidence that DDX3 enhances oncogenic KRAS transcription via an increase in SP1 binding to its promoter. Accelerating oncogenic KRAS expression by DDX3 promotes the invasion capability via the ERK/PTEN/AKT/β-catenin cascade. Moreover, the β-catenin/ZEB1 axis is responsible for DDX3-induced cell invasiveness and xenograft lung tumor nodule formation. The xenograft lung tumor nodules induced by DDX3-overexpressing T84 stable clone were nearly suppressed by the inhibitor of AKT (perifosine) or β-catenin (XAV939). Among patients, high KRAS, positive nuclear β-catenin expression and high ZEB1 were more commonly occurred in high-DDX3 tumors than in low-DDX3 tumors. High-DDX3, high-KRAS, positive nuclear β-catenin tumors, and high-ZEB1 exhibited worse overall survival (OS) and relapse free survival (RFS) than their counterparts. In conclusion, DDX3 may play an oncogenic role to promote tumor growth and invasion in colon cancer cells via the β-catenin/ZEB1 axis due to increasing KRAS transcription. We therefore suggest that AKT or β-catenin may potentially act as a therapeutic target to improve tumor regression and outcomes in colorectal cancer patients who harbored high-DDX3 tumors.

## INTRODUCTION

Mutations of the K-*ras* (KRAS) oncogene are observed in up to 50% of colorectal cancerat the stage of the adenomatous precursors [1]. A two-step model for colon adenoma initiation is caused by adenomatous polyposis coli (APC) mutation as the first step, whereas KRAS activation and β-catenin nuclear localization promote adenoma progression to adenocarcinoma as a second step [2]. This finding supported by a previous study and indicated that KRAS mutation is not necessary for β-catenin activation in APC-familiar adenomatous polyposis (FAP)-associated adenomas [3], but synergistically promotes APC loss-induced tumor progression in colorectal cancer [4]. However, the underlying mechanism of the cross-talk between oncogenic KRAS and APC mutation in colorectal tumorigenesis is not fully understood.

Oncogenic KRAS may be required to maintain in cytoskeletal organization, adhesion, and motility in colon cancer cells, and suggested that mutated KRAS oncogenes are essential for maintenance of invasive phenotype in colon cancer cells [5]. Oncogenic KRAS increased the levels of nuclear β-catenin and formation of nuclear β-catenin/Transcription factor 4 (TCF4) complex in colon cancer through inhibition of Glycogen synthase kinase-3β (GSK-3β), and these effects were blocked by the inhibitor of the phosphatidylinositide 3-kinases (PI3K)/AKT signaling pathway [6]. Expression of KRAS^G12D^ in the colonic epithelium in a transgenic mouse model stimulates hyper-proliferation in a MEK-dependent manner [7]. Inhibition of miR-193a expression by Max and retinoid X receptor alpha (RXRα) activates KRAS expression to promote colorectal tumor growth [8]. Interestingly, ZNF312b promotes the progression of gastric tumor by transcriptional activation of the KRAS gene [9]. These results suggest that an increase in oncogenic KRAS expression may synergistically promote APC loss-induced colorectal tumorigenesis via the β-catenin/TCF4, MEK/ERK, and PI3K/AKT signaling pathways.

DDX3, a DEAD-box RNA helicase has been identified as a regulator of the β-catenin/TCF signaling to act as a regulatory subunit of casein kinase 1ε (CK1ε) to promote phosphorylation of the scaffold protein disheveled, and suggested that DDX3 is required for β-catenin activation during the development of normal mammalian cells [10]. We here provided evidence that DDX3 enhances KRAS transcription via increased SP1 binding to the KRAS promoter. Accelerating oncogenic KRAS expression is responsible for DDX3-induced tumor invasion via the β-catenin/zinc finger E-box binding homeobox 1 (ZEB1) axis. Patients with high-DDX3, high-KRAS, and high-β-catenin tumors exhibited poorer overall survival (OS) and relapse free survival (RFS) than their counterparts.

## RESULTS

### DDX3 promotes cell invasion in KRAS-mutated colon cancer cells

**Figure 1:**
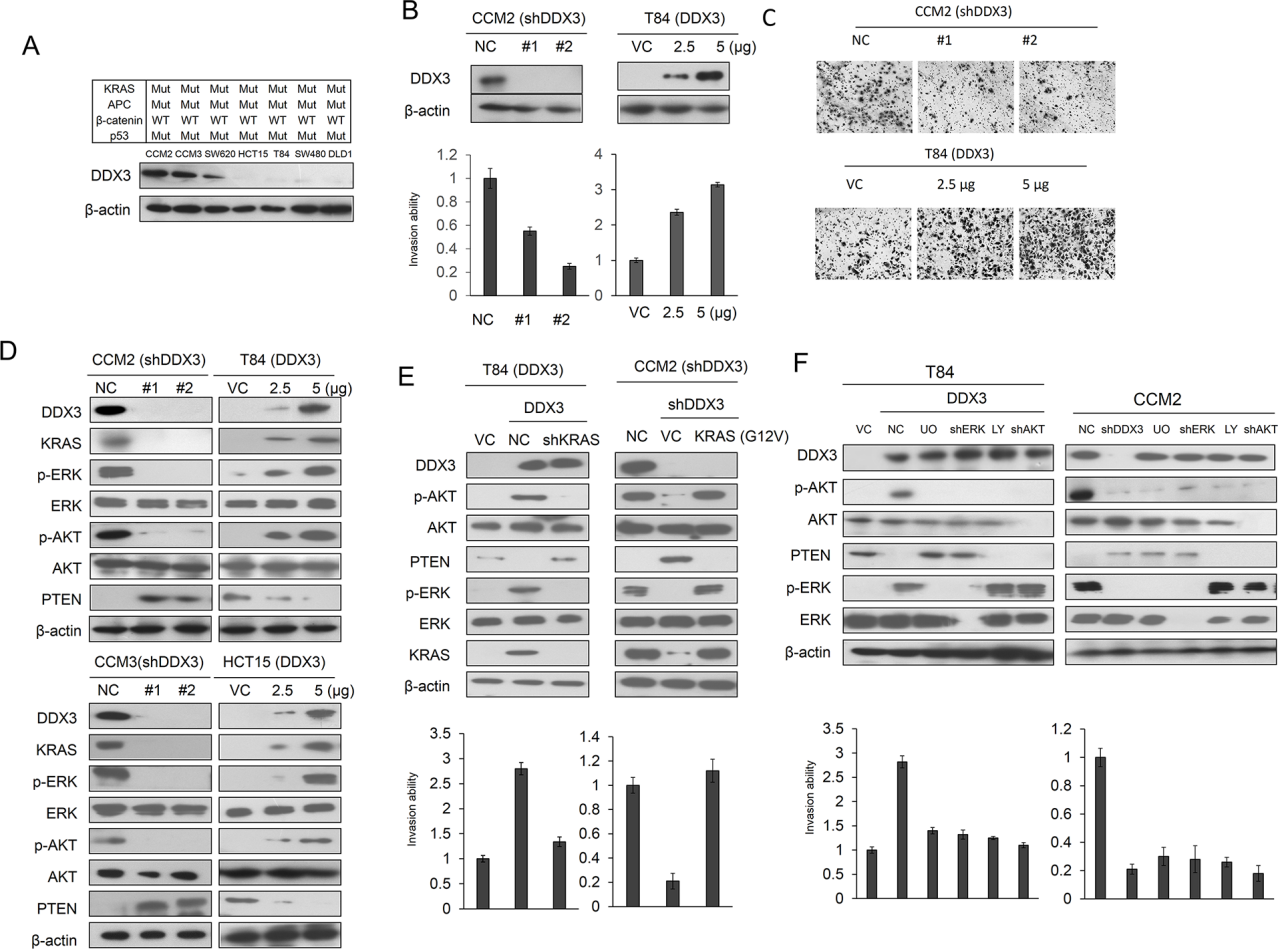
DDX3 promotes cell invasion via the KRAS/ERK/PTEN/AKT cascade (**A**) Seven colon cancer cell lines were enrolled to evaluate the expression levels of DDX3 by western blotting. (**B**) Two kinds of DDX3 shRNA were transfected into high DDX3 expressing CCM2 cells. Two doses of DDX3 expression vector were transfected into low DDX3 expressing T84 cells. After 48 hr, the lysates were harvested and evaluated for levels of DDX3 and β-actin protein by Western blotting. The invasion capability were evaluated in CCM2 cells with or without shDDX3 transfection and in T84 cells with or without DDX3 expression vector transfection and the efficacy of invasion ability were compared with NC and VC. (**C**) Representative invasive cells are shown on Matrigel membranes. (**D**) The expression of KRAS, p-ERK, p-AKT, ERK, AKT and PTEN in DDX3-knockdown CCM2 and CCM3 cells and DDX3-overexpression T84 and HCT15 cells were evaluated by western blotting. (**E**) T84 cells were transfected with indicated combination of DDX3 expression vector and KRAS shRNA for 24 hr. CCM2 cells were transfected with indicated combination of DDX3 shRNA, mutant KRAS (G12V) for 24 hr. The invasion capability was evaluated by Boyden chamber assay for additional 16 hr. The expression of DDX3, KRAS, p-ERK, p-AKT, ERK, AKT and PTEN was determined by Western blotting. (**F**) T84 cells were transfected with indicated combination of DDX3 expression vector, ERK shRNA and AKT shRNA for 24 hr. CCM2 cells were transfected with DDX3, ERK and AKT shRNA for 24 h. After 24 hr, these cells were treated with or without 10 μM LY294002 and U0126 for 5 hr. The invasion capability was evaluated by Boyden chamber assay for additional 16 hr.

We examined whether DDX3 could synergistically enhance APC loss-induced cell invasion in KRAS-mutated cells. Seven colon cancer cell lines harbored KRAS, APC, and p53 mutations, but possessed wild-type β-catenin gene were enrolled to test the possibility. Western blotting analysis showed that DDX3 was highly expressed in CCM2 and CCM3 cells followed by SW620, HCT15, T84, SW480, and DLD1 cells (Figure 1A). The high expressing CCM2 and CCM3 cells and the low expressing T84 and HCT15 cells were transfected with DDX3 shRNA (shDDX3) and its expression vector respectively. The invasion capability decreased significantly in DDX3-knockdown CCM2 cells, but increased in DDX3-overexpressing T84 cells (Figure 1B). The representative invasive cells on matrigel membranes are shown in Figure 1C. These results suggest that DDX3 may enhance invasion capability in colon cancer cells.

### DDX3 promotes cell invasion via the KRAS/ERK/PTEN/AKT cascade

Oncogenic RAS suppresses the apoptotic gene PTEN via the RAF/MEK/ERK/c-Jun pathway to induce anti-apoptosis and cellular transformation [11]. We therefore examined the possibility that DDX3 could activate the MEK/ERK and the PI3K/AKT signaling pathways to promote invasion capability in KRAS-mutated colon cancer cells. The CCM2, CCM3, T84, and HCT15 cells were selected to manipulate DDX3 gene expression by shDDX3 and its expression vector. Unexpectedly, KRAS expression was markedly decreased by DDX3 knockdown in CCM2 and CCM3 cells, but was increased by DDX3 overexpression in T84 and HCT15 cells (Figure 1D). The expression levels of p-ERK and p-AKT expression were elevated and reduced by DDX3 manipulation in these cell types. The p-AKT expression was negatively correlated with the change of PTEN expression by DDX3 manipulation (Figure 1D). PTEN reduction by ERK activation in T84 cells was consistent with a previous study [11] (Supplementary Figure S1). The invasion capability were markedly decreased by KRAS silencing in DDX3-overexpressing T84 cells, but were increased by ectopic KRAS expression in DDX3-knockdown CCM2 cells (Figure 1E). The increase of invasion capability in DDX3-overexpressing T84 cells can be reversed by ERK or AKT inhibitor (U0126 or LY294002) treatment or shERK or shAKT transfection. The invasion capability was suppressed by shDDX3, shERK, shAKT transfection or U0126 and LY294002 treatment (Figure 1F). These results suggest that DDX3-induced cell invasion may be through the KRAS/ERK/PTEN/AKT cascade.

### DDX3 enhances KRAS transcription via increased SP1 binding to the KRAS promoter

**Figure 2:**
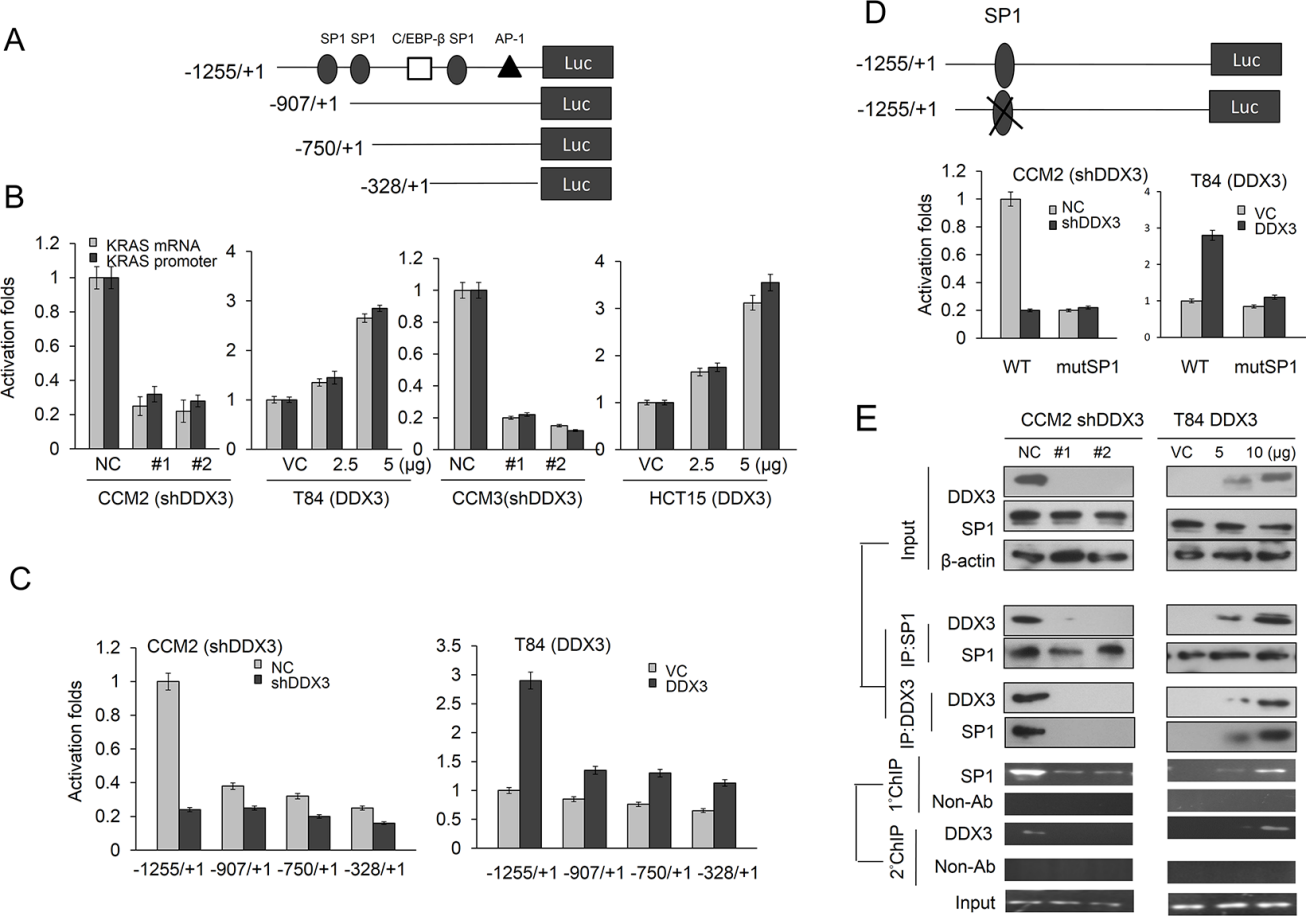
DDX3 enhances KRAS transcription via increased SP1 binding to the KRAS promoter (**A**) Schematic diagram of KRAS-promoter-driven luciferase reporters: KRAS (−1255/+1)-Luc, KRAS (−907/+1)-Luc, KRAS (−750/+1)-Luc, and KRAS (−328/+1)-Luc. (**B**) KRAS mRNA and promoter (−1255/+1) activity in DDX3-knockdown CCM2 and CCM3 cells and DDX3-overexpression in T84 and HCT15 cells was evaluated by real-time PCR and luciferase reporter activity assay. (**C**) The KRAS promoter constructs and shDDX3 or its expression vector was co-transfected into the indicated cell types. Luciferase reporter activity was measured at 48 hr after post-transfection. (**D**) Schematic diagram of KRAS-promoter-driven luciferase reporters (−1255/+1) and mutated construct of SP1 binding site. The two KRAS promoter constructs and shDDX3 or its expression vector were co-transfected into the indicated cell types. Luciferase reporter activity was measured at 48 hr after post-transfection. (**E**) CCM2 cells transfected with two kinds of shDDX3 and T84 cells were transfected with DDX3expression vector for 48 hr. Both cell lysates were immunoprecipitated with anti-SP1-conjugated beads. The immunoprecipitates were analyzed by SDS–PAGE, followed by immunoblotting with anti-DDX3 antibody. The reciprocal experiment was performed using the anti-DDX3 antibody for immunoprecipitations followed by western blot with anti-SP1. The input control was 30% of the cell extract without any treatment. Binding activity of SP1 to the KRAS promoter was evaluated by ChIP. Chromatin was isolated and immunoprecipitated with an antibody specific for SP1. For ChIP-reChIP assays, crosslinked protein–DNA complexes were eluted from primary immunoprecipitates by incubation with 10 mM dithiothreitol for 30 min at 37°C. The eluates were diluted 1:50 in dilution buffer and then subjected to immunoprecipitation with the secondary antibodies.

DDX3 has been shown to promote SP1 binding to p21 and MDM2 promoter to increase both gene expressions [12, 13]. We therefore hypothesized that an increase in KRAS expression by DDX3 could be through enhancing KRAS transcription via increased SP1 binding to the KRAS promoter. The KRAS promoter (−1255/+1) containing three SP1 putative binding site at −945/−939, −905/−898, and −322/−316 was constructed by PCR to test the possibility (Figure 2A). Luciferase reporter assay and real-time PCR analysis indicated that the −1255/+1 KRAS promoter activity and its mRNA expression levels were significantly reduced by DDX3-knockdown in CCM2 and CCM3 cells, but were elevated by DDX3-overexpression in T84 and HCT15 cells (Figure 2B). To verify whether the SP1 binding site located at the −1255/+1 KRAS promoter could be involved in DDX3-induced KRAS transcription, the three short KRAS promoters (−907/+1, −750/+1 and −328/+1) were constructed by PCR and deletion mutation (Figure 2A). Luciferase reporter assay revealed that the −1255/+1 KRAS promoter activity was significantly elevated by DDX3 overexpression, but the −750/+1 and −328/+1 KRAS promoter activity were almost unchanged by DDX3 overexpression in T84 cells (Figure 2C). The −1255/+1 KRAS promoter was markedly decreased and increased by DDX3-knockdown and –overexpression in CCM2 and T84 cells, but the −907/+1, −750/+1 and −328/+1 KRAS promoter activity was almost unchanged by DDX3 manipulation in both cell types (Figure 2C). In addition, the promoter activity were also unchanged by transfecting the −1255/+1 KRAS promoter, which harbored a mutant SP1 binding site, into DDX3-overexpressing T84 and DDX3-knockdown CCM2 cells (Figure 2D). Western blotting and immunoprecipitation (IP) showed that the interaction of DDX3 with SP1 was significantly increased in DDX3-overexpressing T84 cells, but was decreased in DDX3-knockdown CCM2 cells (Figure 2E). ChIP and ChIP-reChIP further indicated that the binding activity of SP1 onto the −1255/+1 KRAS promoter was dose-dependently increased in DDX3-overexpressing T84 cells (Figure 2E right panel) and the opposite was observed in DDX3-knockdown CCM2 cells (Figure 2E left panel). These results clearly indicated that KRAS transcription can be enhanced by DDX3 overexpression via increased SP1 binding to the KRAS promoter.

### An increase in β-catenin expression promotes DDX3-mediated invasiveness via activation of the PI3K/AKT signaling pathway due to accelerating KRAS expression

**Figure 3:**
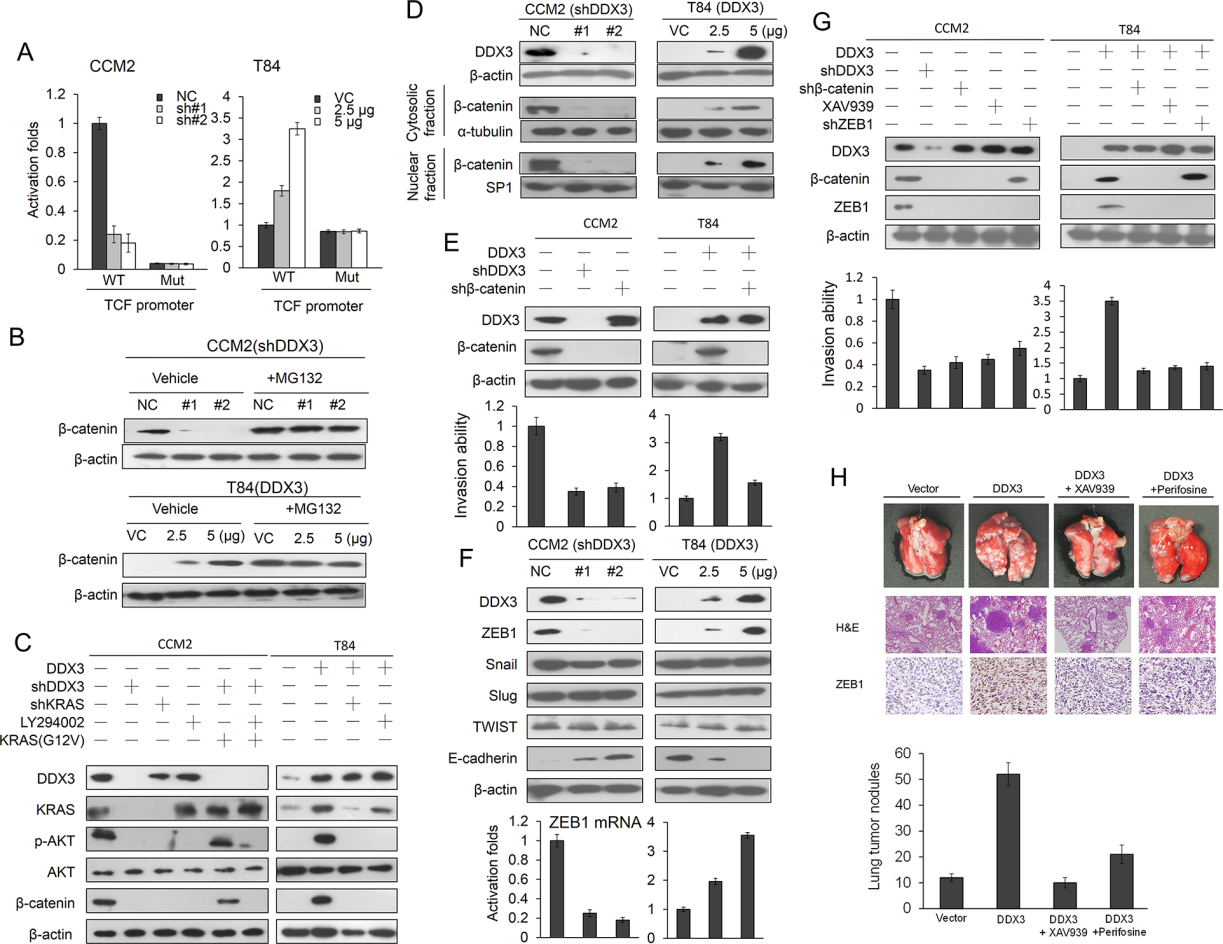
DDX3-mediated β-catenin activation is responsible for cell invasion via increasing ZEB1 expression (**A**) The TCF promoter activity (TOP flash, WT and FOP flash, Mut) in DDX3-knockdown in CCM2 cells and DDX3-overexpressing T84 cells was evaluated by luciferase reporter assay. (**B**) CCM2 and T84 cells were transfected with two kinds of DDX3 shRNA and two doses of DDX3 expression vector. These cells were incubated with 5 μM of MG132 for an additional 5 hr, and then the cell lysates were analyzed by SDS-PAGE, followed by immunoblotting with anti-β-catenin antibody. (**C**) CCM2 cells were co-transfected with the indicated combinations of shDDX3, shKRAS and mutant KRAS (G12V) for 24 hr. T84 cells were co-transfected with the indicated combinations of DDX3 and shKRAS Consequent, these cells were treated with or without 10 μM LY294002. After treatment for 4 hr, the cells lysates were separated by SDS-PAGE for the protein expression using western blotting. (**D**) CCM2 and T84 cells were transfected with DDX3 shRNA and its expression vector for 48 hr. These cells were separated with cytosolic and nuclear fraction to evaluate β-catenin protein expression by SDS-PAGE using a western blotting. (**E**) CCM2 cells were transfected with shDDX3 or shβ-catenin for 24 hr. T84 cells were transfected with DDX3 expression vector or combined with shβ-catenin transfection for 24 hr. The expression of DDX3 and β-catenin in these treated both cells were evaluated by western blotting and the invasion capability was determined by Boyden chamber assay. The efficacy of invasion ability was compared with NC and VC, respectively. (**F**) Two kinds of shDDX3 were transfected into high DDX3 expressing CCM2 cells and two doses of DDX3 expression vector were transfected into low DDX3 expressing T84 cells. After 48 hr, the cell lysates were used for evaluating the expression of DDX3, ZEB1, Snail, Slug, and TWIST by Western blotting. ZEB1 mRNA expression was evaluated by real-time PCR. (**G**) CCM2 cells were transfected with shDDX3, shβ-catenin, shZEB1 and β-catenin inhibitor (XAV939) singly or in combination, for 24 hr. T84 cells were transfected with DDX3 expression vector, β-catenin, ZEB1-knockdown plasmids, and β-catenin inhibitor (XAV939) singly or in combination, for 24 hr. The invasion capability in both cells with different treatments were evaluated by Boyden chamber assay for 16 hr. (**H**) Example of lungs of mice with visual lung tumor nodules at 6 weeks after tail vein inoculation of indicated cells. Representative Hematoxylin and Eosin staining was presented in lung tumor nodules from each group of mice. The number of lung tumor nodules of each group of mice. Data are presented as mean ± SD. ZEB1 expressions in lung tumors were evaluated by IHC using a specific antibody.

The TOP flash (a reporter plasmid containing wild-type TCF-binding site) and FOP flash (a reporter plasmid containing mutant TCF-binding site) plasmids was used to verify whether the transcriptional activity of β-catenin was modulated by DDX3 manipulation in colon cancer cells. Luciferase reporter assay showed that the TOP flash was decreased in DDX3-knockdown CCM2 cells and was increased in DDX3-overexpressing T84 cells in a dose-dependent manner. However, the FOP flash was unchanged in DDX3 manipulation in both cell types (Figure 3A). Western blotting indicated that β-catenin expression level decreased markedly in DDX3-knockdown CCM2 and increased in DDX3-overexpressing T84 cells. Western blotting showed that the disappearance of β-catenin expression in DDX3-knockdown CCM2 and T84 NC cells can be reversed by MG132 treatment (Figure 3B). In the presence of MG132, β-catenin ubiquitin pattern evaluated by immunoprecipitation (IP) and western blotting was more revealed in DDX3-knockdown CCM2 and in T84 VC cells when compared with CCM2 cells transfecting with non-specific shRNA (NC) and DDX3-overexpressing T84 cells (Supplementary Figure S2A). The half-life of β-catenin expression was significantly reduced in parental T84 cells and DDX3-knockdown CCM2 cells compared with DDX3-overexpressing T84 cells and parental CCM2 cells (Supplementary Figure S2B). Western blotting revealed that the expression of KRAS and β-catenin was eliminated by DDX3 or KRAS silencing, but KRAS expression was unchanged by LY294002, shDDX3 plus KRAS or shDDX3, LY294002 plus KRAS treatments in CCM2 cells (Figure 3C). However, β-catenin expression was reversed by shDDX3 plus KRAS transfection in CCM2 cells (Figure 3C). Conversely, β-catenin expression was markedly increased by DDX3 overexpression in T84 cells, but its expression was reversed by DDX3 plus shKRAS or DDX3 plus LY294002 treatments (Figure 3C). These results suggested that an increase in β-catenin expression by DDX3 overexpression is through activation of the PI3K/AKT pathway due to increasing KRAS expression. In addition, an increase in β-catenin expression by DDX3 overexpression was correlated with β-catenin nuclear localization in colon cancer cells (Figure 3D). These results suggested that an increase in β-catenin expression may be responsible for DDX3-mediated cell invasiveness via the PI3K/AKT signaling pathway due to accelerating KRAS expression.

### The β-catenin/ZEB1 axis is responsible for DDX3-mediated cell invasion

The β-catenin/TCF4 complex induces the epithelial-to-mesenchymal transition (EMT)-activator ZEB1 to promote tumor invasion in APC-mutated colon cancer [14]. We therefore expected that upregulation of ZEB1 by β-catenin activation could be responsible for DDX3-induced cell invasion. The invasion capability decreased markedly in DDX3-knockdown or β-catenin-knockdown in CCM2 cells when compared with their NC cells (Figure 3E left upper panel). The increase of invasive capability by DDX3 overexpression in T84 cells was nearly eliminated by β-catenin silencing (Figure 3E right upper panel). Western blotting showed that ZEB1 expression decreased in DDX3-knockdown CCM2 cells and increased markedly in DDX3-overexpressing T84 cells. However, the expression of Snail, Slug, and TWIST was unchanged by DDX3 manipulation in both cell types (Figure 3F upper panel). The modulation of ZEB1 protein expression by DDX3 manipulation in both cell types was consistent with its mRNA expression (Figure 3F lower panel). The decrease of the cell invasion capability was correlated with a decrease in ZEB1 expression by shDDX3, shβ-catenin, shZEB1 or XAV939 in CCM2 cells and DDX3-overexpressing T84 cells (Figure 3G). These results clearly indicate that the β-catenin/ZEB1 axis may be responsible for DDX3-induced cell invasion.

We further examined whether DDX3-induced tumor invasion could be observed in the animal model. The lung tumor nodules were counted in nude mice injected with DDX3-overexpressing T84 stable clone via tail vein compared with nude mice injected with VC cells (Figure 3H upper panel). The histology of lung tumors from each group's mice was confirmed by H & E staining (Figure 3H lower panel). ZEB1 expression was significantly decreased by an AKT inhibitor (perifosine) or a β-catenin inhibitor (XAV939) in tumors of mice injected with DDX3-overexpressiong T84 stable clone (Figure 3H lower panel). The number of lung tumor nodule in mice injected with DDX3-overexpressing T84 stable clone was markedly higher than in mice injected with VC cells. However, the number of lung tumor nodule in mice injected with DDX3-overexpressing T84 stable clone was significantly suppressed by perifosine or XAV939 treatment. These results obtained from the animal model strongly support the mechanistic action in the cell model, and indicated that the β-catenin/ZEB1 axis may be responsible for DDX3-induced cell invasion and xenograft lung tumor formation.

### DDX3 expression is positively correlated with KRAS and nuclear β-catenin expression in tumor tissues and associated with OS and RFS in colorectal cancer patients

**Figure 4:**
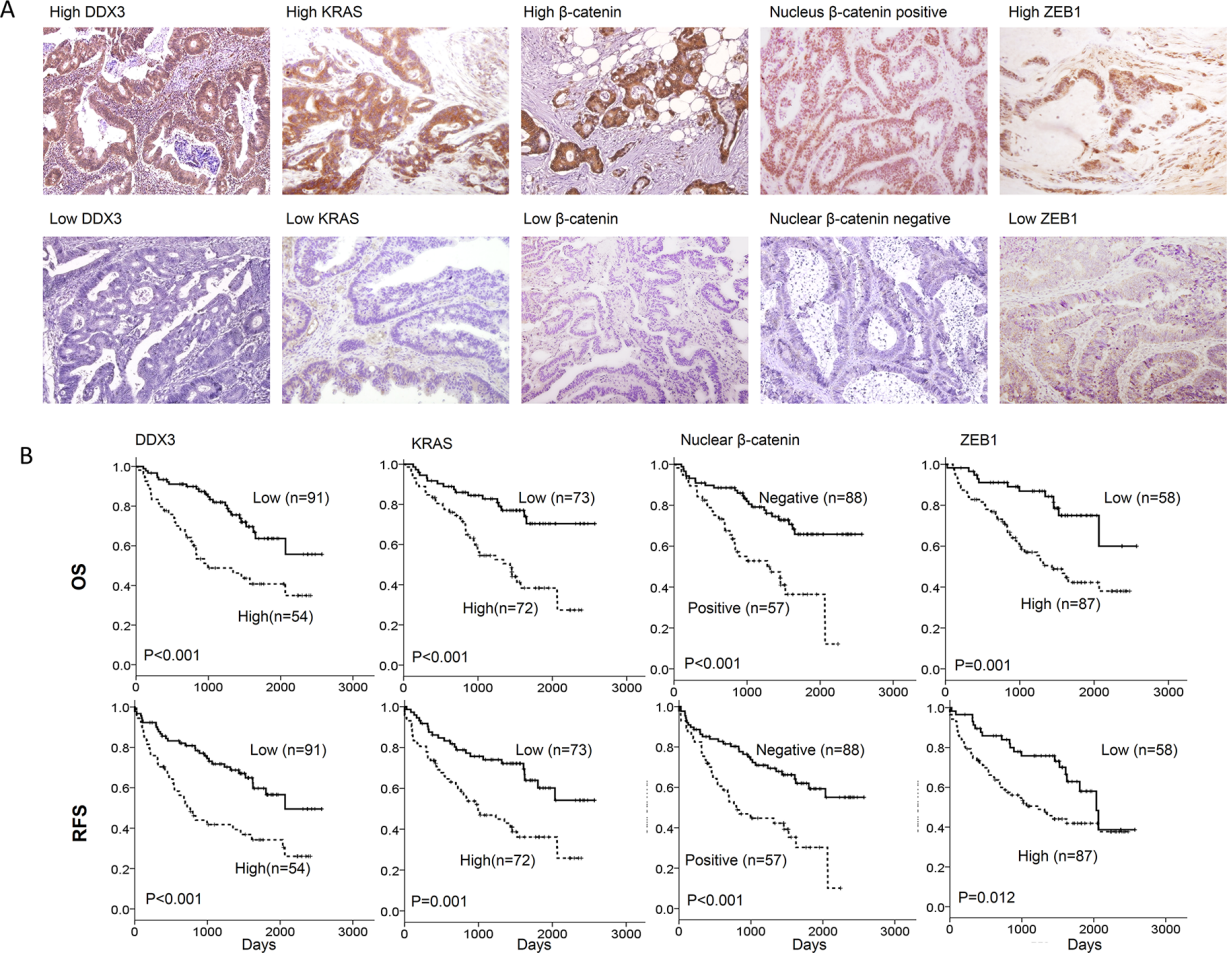
The representative immunostaining results of DDX3, KRAS, β-catenin expression in nucleus/cytoplasm, and nuclear β-catenin in colorectal tumors and the prognostic value of these molecules on OS and RFS was assessed using a Kaplan-Meier analysis (**A**) The representative immunostaining results of DDX3, KRAS, ZEB1, β-catenin, and nuclear β-catenin in colorectal tumors; (**B**) The survival curves in colorectal patients with high-DDX3, high-KRAS, positive nuclear β-catenin tumors and high-ZEB1 for OS and RFS were compared to those with low-DDX3, low-KRAS, negative nuclear β-catenin, and low-ZEB1 tumors.

We examined the possibility that DDX3 expression could be correlated with KRAS, β-catenin and ZEB1 expression in tumors and their expressions could be associated with clinical outcomes in colorectal cancer patients, 145 tumors were enrolled to evaluate DDX3, KRAS, β-catenin, and ZEB1 expression by IHC. The representative immunostaining results of DDX3, KRAS, β-catenin, and ZEB1 are shown in Figure 4A. DDX3 expression was not associated with clinical parameters in this study population including age, genders, smoking status, and stage (data not shown). High KRAS, high β-catenin, positive nuclear β-catenin and high ZEB1 expression were more commonly observed in high-DDX3 tumors than in low-DDX3 tumors (*P* = 0.002 for KRAS, *P* < 0.001 for β-catenin, *P* < 0.001 for nuclear β-catenin, *P* = 0.008 for ZEB1; Table 1). Positive nuclear β-catenin, high β-catenin or high ZEB1 expression was more prevalent in high-KRAS tumors than in low-KRAS tumors (*P* < 0.001 for β-catenin, *P* = 0.001 for nuclear β-catenin, *P* = 0.001 for ZEB1; Table 1). The positive correlation of β-catenin with positive nuclear β-catenin and high ZEB1 expression was also revealed in this study population (*P* < 0.001 for nuclear β-catenin, *P* = 0.002 for ZEB1; Table 1).

**Table 1:** The correlation of DDX3 with KRAS, β-catenin, nuclear β-catenin, and ZEB1 expression and their each correlation in colorectal cancer patients

		KRAS			β-catenin			Nuclear β-catenin			ZEB1		
	No	Low	High	*P*	Low	High^a^	*P*	Negative	Positive^b^	*P*	Low	High	*P*
**DDX3**													
Low	91	55 (60)	36 (40)	0.002	45 (50)	46 (50)	< 0.001	69 (76)	22 (24)	< 0.001	44 (48)	47 (52)	0.008
High	54	18 (33)	36 (67)		6 (11)	48 (89)		19 (35)	35 (65)		14 (26)	40 (74)	
**KRAS**													
Low	73				35 (48)	38 (52)	0.001	55 (75)	18 (25)	< 0.001	39 (53)	34 (47)	0.001
High	72				16 (22)	56 (78)		33 (46)	39 (54)		19 (26)	53 (74)	
**β-catenin**													
Low	51							51 (100)	0 (0)	< 0.001	31 (61)	20 (39)	< 0.001
High	94							37 (39)	57 (61)		27 (29)	67 (71)	
**Nuclear β-catenin**													
Negative	88										44 (50)	44 (50)	0.002
Positive	57										14 (25)	43 (75)	

a The high expression for β-catenin was including with the expression of β-catenin in cytoplasm or nucleus.

b The positive expression for β-catenin was including with the expression of β-catenin in nucleus.

Kaplan-Meier analysis showed that high-DDX3, high-KRAS, positive nuclear β-catenin, and high-ZEB1 tumors exhibited shorter OS and RFS periods than low-DDX3, low-KRAS, negative nuclear β-catenin, and low-ZEB1 tumors (Figure 4B). Cox-regression analysis further indicated that the hazard ratio of high-DDX3, high-KRAS, positive nuclear β-catenin, high-ZEB1, and high-DDX3/high-ZEB1 tumors was 3.05, 2.72, 2.90, 3.17, and 2.40 for OS and 2.52, 2.21, 2.47, 2.03, and 1.81 for RFS when the counterpart or others was used as the reference (Table 2). The highest HR value for OS and RFS was observed in high-DDX3/high-ZEB tumors when compared with low-DDX3/low-ZEB1 tumors (11.87 for OS, 5.01 for RFS). These results obtained from colorectal cancer patients may support the findings in the cell and animal model, and indicated that activation of the β-catenin/ZEB1 axis by DDX3 overexpression may promote tumor invasion and consequently result in poorer outcome in colorectal cancer patients.

**Table 2:** Cox regression analysis of DDX3, K-ras, nuclear β-catenin, and ZEB1 expressions in patients with colorectal cancer

	OS				RFS			
Variables	Case No.	Adjusted HR*	95%CI	*P*	Case No.	Adjusted HR*	95%CI	*P*
**DDX3**								
Low	91	1			91	1		
High	54	3.05	1.77–5.24	< 0.001	54	2.52	1.54–4.12	< 0.001
**KRAS**								
Low	73	1			73	1		
High	72	2.72	1.53–4.85	0.001	72	2.21	1.33–3.69	0.002
**Nuclear β-catenin**								
Negative	88	1			88	1		
Positive^a^	57	2.90	1.68–4.99	< 0.001	57	2.47	1.51–4.05	< 0.001
**ZEB1**								
Low	58	1			58	1		
High	87	3.17	1.64–6.13	0.001	87	2.03	1.19–3.48	0.010
**DDX3/ZEB1**								
Others	105	1			105	1		
High/High	40	2.40	1.36–4.22	0.002	40	1.81	1.05–3.11	0.032
**DDX3/ZEB1**								
Low/Low	44	1			44	1		
High/High	40	11.87	3.44–41.0	< 0.001	40	5.01	2.15–11.69	< 0.001

OS: overall survival; HR: Hazard ratio; RFS: relapse-free survival.*HR adjusted for age, gender, smoking staus, and duke stage.

a The positive expression for β-catenin was including with the expression of β-catenin in nucleus.

## DISCUSSION

The involvement of DDX3 in human tumorigenesis plays a tumor suppressor gene in hepatocellular carcinoma and lung cancer [12, 13, 15, 16], but acts as an oncogene in breast cancer [17, 18]. Recently, DDX3 was shown to promote invasive behavior via Rac1-mediated Δ-catenin activation in cervical cancer and neuroblastoma cells [19]. Inhibition of DDX3 expression by its shRNA resulted in reduced proliferation and a G1-arrest in HCT116 and HT29 colon cancer cells [20]. High DDX3 expression was positively correlated with nuclear β-catenin expression in tumors from colorectal cancer patients [20]. These results support our findings to suggest that DDX3 plays an oncogenic role in colorectal tumor invasion. However, a recent report indicated that DDX3 as a tumor suppressor role to inhibit tumor metastasis and exhibiting a favorable prognosis in colorectal cancer [21]. A relatively lower expression of DDX3 in DLD1 colon cancer cells were transfected with shRNAs, particularly a low transfection efficiency of shRNA (~50%), seemed to be not really reflected the effects of DDX3 on cell migration and invasion. In addition, no data supported DDX3 as a tumor suppressor by transfection of DDX3 expression vector in colon cancer cells. Therefore, the conflicting role of DDX3 in patients' prognosis between Su's and our present study is needed to further verify.

We provide evidence to demonstrate that DDX3 may promote tumor invasion in APC-mutated colon cancer cells via the β-catenin/ZEB axis. We questioned whether DDX3 could activate the β-catenin/ZEB axis in APC-wild-type colon cancer cells. APC-mutated T84 cells were used to knockdown APC and then co-transfected with a wild-type APC expression vector. As expected, the TCF promoter activity, β-catenin and ZEB1 protein expressions increased markedly in DDX3-overexpressing T84 cells compared with VC cells (Supplementary Figure S3). The elevation of the TCF promoter activity, β-catenin, and ZEB1 expressions was still elevated by DDX3 overexpression in APC-knockdown T84 cells. However, the TCF promoter activity and both protein expressions in APC-knockdown T84 cells was significantly reduced by APC-wild-type expression vector transfection, but the decrease of the TCF promoter activity and both protein expressions were relatively elevated by DDX3 overexpression compared with its VC cells (Supplementary Figure S3). We further examined whether DDX3 could also activate the β-catenin/ZEB1 axis in HCT116 cells which harbored β-catenin mutation and wild-type APC. Western blotting indicated that the expression of β-catenin and ZEB1 was markedly decreased by DDX3-knockdown, but both protein expressions were increased by DDX3 overexpression in HCT116 cells in a dose-dependent manner (Supplementary Figure S4). These results suggest that DDX3 promoted cell invasion via the β-catenin/ZEB axis in colorectal cancer, regardless of APC and β-catenin mutations.

The AKT/GSK3β signaling pathway promoted nuclear β-catenin expression via reducing N-terminal β-catenin phosphorylation (Ser33, Ser37, Thr41), which is associated with degradation of β-catenin [22]. AKT-mediated β-catenin signaling activation is required for progenitor cell activation during the progression from chronic ulcerative colitis to colitis-associated cancer [23]. ERK activation by HBV-X protein has been shown to increase β-catenin expression via phosphorylation of GSK3β at Serine (Ser) 9 [24]. However, phosphorylation of GSK3β at Ser9 is concomitantly through the RAS/MEK/ERK and AKT signaling to independently induce phosphorylation of GSK3β at Ser9 [24, 25]. In current study, the increase of β-catenin expression by phosphorylation of GSK3β at Ser9 due to PI3K/AKT activation by DDX3 overexpression revealed in CCM2 and T84 cells (Supplementary Figure S5). Therefore, DDX3-induced tumor invasion due to increasing β-catenin expression by its protein phosphorylation and stability via the KRAS/ERK/PTEN/AKT/GSK3β cascade.

In summary, we provide evidence that DDX3 enhances KRAS transcription to activate the β-catenin/ZEB1 axis via the ERK/AKT signaling pathway. In addition, the β-catenin/ZEB1 axis is responsible for DDX3-induced invasiveness in colon cancer cells, lung tumor nodule formation in nude mice, and poor outcomes in colorectal cancer patients. Therefore, we suggest that AKT or β-catenin may be potentially targeted to suppress tumor invasion and in turn to improve outcomes in patients who harbored high-DDX3 tumors.

## MATERIALS AND METHODS

### Study subjects

This study enrolled of 145 patients with colorectal cancer. The inclusion criteria for patients were: primary diagnosed with colorectal carcinoma; no metastatic disease at diagnosis; no previous diagnosis of carcinoma; no neoadjuvant treatment before primary surgery; no evidence of disease within one month of primary surgery. Tumor specimen collected from surgically-resected colorectal cancer patients were stored at 80°C in the Division of Colon and Rectum, Chung Shan Medical University Hospital (Taichung, Taiwan), between 1994 and 2006. Patients were asked to submit written informed consent and the study was approved by the Institutional Review Board (CS07159). The tumor stage of each specimen were histologically determined according to the WHO classification system. Cancer relapse data were obtained by chart review and confirmed by surgeons. Clinical parameters and overall survival (OS) data were collected from chart review and the Taiwan Cancer Registry, Department of Health, Executive Yuan, Taiwan, ROC. Survival time was defined as the period from the date of primary surgery to the date of death. The median follow-up time was 1432 days (ranging from 102 to 2572 days) and the end of the follow-up period was December 2007. Based on follow-up data, relapse data from 145 patients were available, indicating that 29 patients relapsed (22 had distant metastasis, and 7 had local and distant metastasis). Tumors frequently relapsed in the liver (13 patients), metastasized in the lung (4 patients), hypopharynx (1 case) bone (1 case), left para-aortic lymph node (1 case), pelvic (1 case), rectum (1 case), and 7 patients had tumors that metastasized to more than one organ.

### Cell lines

CCM2 and CCM3 cells were cultured from Taiwanese colorectal cancer patients and kindly provided by Dr. Wun-Shaing Wayne Chang (National Institute of Cancer Research, National Health Research Institutes, Miaoli, Taiwan). All other cells were obtained from the American Type Culture Collection (ATCC) and the culture condition as described elsewhere. Cells were cultured and stored according to the suppliers' instructions and used at passages 5 to 20. Once resuscitated, cell lines are routinely authenticated (once every 6 months, cells were last tested in December 2013) through cell morphology monitoring, growth curve analysis, species verification by isoenzymology and karyotyping, identity verification using short tandem repeat profiling analysis, and contamination checks. The KRAS, APC, β-catenin, and p53 mutation status were shown in Supplementary Table S1.

### Chemicals and antibodies

XAV939 and perifosine were obtained from Selleckchem (Houston, TX, USA). All other chemicals were purchased from Sigma Chemical (St. Louis, MO, USA) unless otherwise indicated. Anti-total ERK, and anti-phosphorylated (p)-ERK antibodies were obtained from Cell Signaling (Danvers, MA, USA). All other antibodies were purchased from Santa Cruz Biotechnology (Dallas, TX, USA).

### Plasmid constructs and transfection

DDX3 (#1 TRCN0000000001; #2 TRCN0000 000003), ERK1/2 (p42 TRCN0000010050; p44 TRCN0000 006150), AKT (TRCN0000010163), KRAS (TRCN00 00033260), β-catenin (TRCN0000314991), JUN (TRCN0 000338221), APC (3′UTR: TRCN0000010297) and ZEB1 (TRCN0000017565) shRNAs were purchased from the National shRNA Core Facility, Academia Sinica, Taiwan, ROC. DDX3, KRAS (G12V) and GSK3β overexpression plasmids were obtained from Addgene Company (Cambridge, MA, USA). TOP flash (a reporter plasmid containing multiple copies of wild-type TCF-binding sites), FOP flash (a reporter plasmid containing mutant TCF-binding sites) plasmids was purchased from Millipore (Billerica, MA, USA). The KRAS-Luc plasmid was constructed by inserting a 1255, 907, 750, 328 bps HindIII/KpnI fragment (spanning the promoter region −1255/+1 related to the translation start site of the human KRAS gene) into a HindIII/KpnI-treated pGL3 vector (Promega, Madison, WI, USA). The SP1-mutated binding site on the KRAS promoter constructs containing multiple-point mutations were constructed by the QuickChange site-directed mutagenesis system (Stratagene, La Jolla, CA, USA). The WT sequence (CCCGCC) for SP1 binding sites on KRAS promoter has been changed to the mutated sequence (CAAGCC). Different concentrations of expression plasmids were transiently transfected into colon cancer cells (1 × 10^6^) using the Turbofect reagent (Glen Burnie, MD, USA). After 48 hr, the cells were harvested and whole cell extracts were assayed in subsequent experiments.

### Western blotting

Equal amounts of protein were separated onto sodium dodecyl sulfate-polyacrylamide gel electrophoresis (SDS-PAGE) gels and then transferred onto a polyvinylidene difluoride membrane (PerkinElmer, Norwalk, CT, USA). After blocking, the membranes were reacted with specific antibody at 4°C overnight, followed by incubation with horseradish peroxidase-conjugated secondary antibody for 1 hr. The blots were observed using an enhanced chemiluminescence kit (PerkinElmer).

### Immunohistochemistry (IHC) analysis

The immunohistochemical procedures and quantification methods were described previously [12]. The intensities of signals were evaluated independently by three observers. Immunostaining scores were defined as the cell staining intensity (0 = nil; 1 = weak; 2 = moderate; and 3 = strong) multiplied by the percentage of labeled cells (0–100%), leading to scores from 0 to 300. A score over 150 was rated as “high” immunostaining, while a score less than 150 was rated as “low” immunostaining.

### RNA isolation and real-time PCR

Total RNA was extracted by homogenization in 1 ml TRIzol reagent, followed by chloroform extraction and isopropanol precipitation. A 3 μg sample of total RNA from colorectal tumor tissues was reverse transcribed using SuperScript II Reverse Transcriptase (Invitrogen Life Technologies) and oligo(dT) 15 primer. The primer sequences for detecting KRAS expression were the forward primers, 5′- AGACAGGAGTGGAGGATGCTTTT-3′, and the reverse primer, 5′- TTCACACAGCCAGGAGTCTTTTC-3′. The primer sequences for detecting ZEB1 expression were the forward primers, 5′- CGCAGTCTGGGTGTA ATCGTAA-3′, and the reverse primer, 5′- CTATAGGAGCC AGAATGGGAAAAG-3′.

### Luciferase reporter assay

Cells were transfected with indicated combination of reporter plasmid with overexpression and knockdown plasmids. Luciferase assays were performed using the Luciferase Reporter Assay System (Promega, Madison, WI, USA) 24 hr after transfection. Normalized luciferase activity was reported as the ratio of luciferase activity/β-galactosidase activity.

### Invasion assay

The Boyden chamber with a pore size of 8 μm was used for cell invasion assay. Cells (1 × 10^4^) in 0.5% serum containing culture medium (HyClone, Ogden, UT, USA) were plated in the upper chamber and 10% fetal bovine serum was added to culture medium in the lower chamber as a chemoattractant. The upper side of the filter was covered with 0.2% Matrigel (Collaborative Research, Boston, MA, USA) diluted in RPMI-1640. After 16 hr, cells on the upper side of the filter were removed and cells that adhered to the underside of membrane were fixed in 95% ethanol and stained with 10% Giemsa dye. The number of invasive cells was counted in the ten contiguous fields.

### Immunoprecipitation (IP) assays

For the IP experiments, cells transfected with plasmids were harvested and cell lysates were prepared using the IP lysis buffer [20 mmol/L Tris-HCl (pH 7.5), 150 mmol/L NaCl, 10% glycerol, and 1% Triton X-100]. Cell extracts (1.5 mg) were incubated with 40 μL of anti-antibody-agarose affinity gel (Millipore). After extensive washing with immunoprecipitation lysis buffer, the immunoprecipitated proteins were analyzed by immunoblotting using specific antibodies

### Chromatin immunoprecipitation (ChIP) assay

Immunoprecipitated DNA was precipitated with ethanol and resuspended in 20 μL ddH2O (double distilled water). For ChIP-reChIP assays, crosslinked protein–DNA complexes were eluted from primary immunoprecipitates by incubation with 10 mm dithiothreitol for 30 min at 37°C. The eluates were diluted 1:50 in dilution buffer and then subjected to immunoprecipitation with the second antibodies. PCR amplification of immunoprecipitated DNA was carried out using the primers consisting of the oligonucleotides that encompass the promoter region of KRAS. The PCR products were separated on 2% agarose gels and analyzed using ethidium bromide staining. The primer sequence of the SP1 binding site on the KRAS promoter was: the forward primer, 5′- AGTAGAGAGAGGGTTTCACCAT-3′ and the reverse primer, 5′- TCATCCTGTAATCCCAGCCTTT-3′.

### *In vivo* tail-vein injection animal model

The *tail*-*vein injection* was according with previous studies [26–28]. The procedures of therapeutic experiments were modified according to previous reports [29, 30]. Mice were injected intraperitoneally with vehicle control (Saline), XAV939 (5 mg/kg), perifosine (5 mg/kg) (*n* = 5, per group) daily from day 1 to day 14. Mice were injected with T84 VC and DDX3-overexpression stable clone via the tail vein (10^5^ cells in 0.1 mL of PBS) at day 7. Six weeks after injection of tumor cells, mice were euthanized, and lungs were dissected and examined for the development of visible lung tumor nodules. Tissues were either processed for Hematoxylin and Eosin staining. These animals were maintained in individual ventilated cages according to the guidelines established in “Guide For The Care and Use of Laboratory Animals” prepared by the Committee on Care and Use of Laboratory Animals of the Institute of Laboratory Animal Resources Commission on Life Sciences, National Research Council, U.S.A. (1985). Use of animals has been approved by the Institutional Animal Care and Use Committee of Taipei Medical University, Taipei, Taiwan (LAC-2013-0205).

### Statistical analysis

Statistical analysis was performed using the SPSS statistical software program (Version 18.0; SPSS Inc., USA). The association between DDX3, KRAS, β-catenin protein and ZEB1 expression was analyzed by the chi-square test. Survival plots were generated using the Kaplan-Meier method and differences between patient groups were determined by the log-rank test. Multivariate Cox regression analysis was performed to determine OS and RFS. The analysis was stratified for all known variables (age, gender, smoking status and tumor stage) and protein expression.

## SUPPLEMENTARY MATERIALS FIGURES AND TABLES


