# Supplementary material for: DDX3 enhances oncogenic KRAS-induced tumor invasion in colorectal cancer via the β-catenin/ZEB1 axis
Source: Oncotarget. 2016 Mar 17;7(16):22687–99. doi: 10.18632/oncotarget.8143 (PMC5008392; doi:10.18632/oncotarget.8143)
Supplement: Supplementary file 1 [file oncotarget-07-22687-s001.pdf]

DDX3 enhances oncogenic KRAS-induced tumor invasion in colorectal cancer via the β-catenin/ZEB1 axis

Supplementary Materials

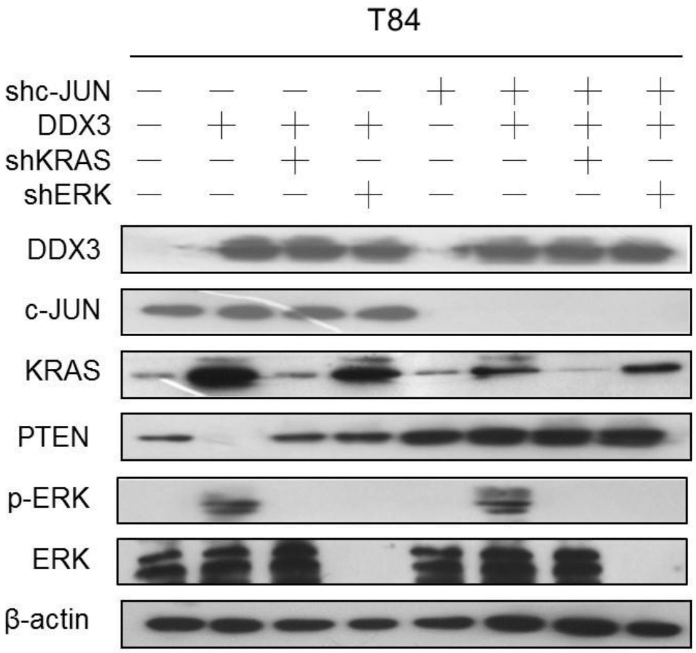

Supplementary Figure S1: PTEN reduction was modulated by DDX3 overexpression via KRAS/ERK/c-JUN axis. T84 cells were transfected with indicated combination of DDX3 expression vector and KRAS, c-JUN, ERK shRNA for 48 hr. The expression of DDX3, KRAS, p-ERK, p-AKT, ERK, AKT and PTEN was determined by Western blotting.

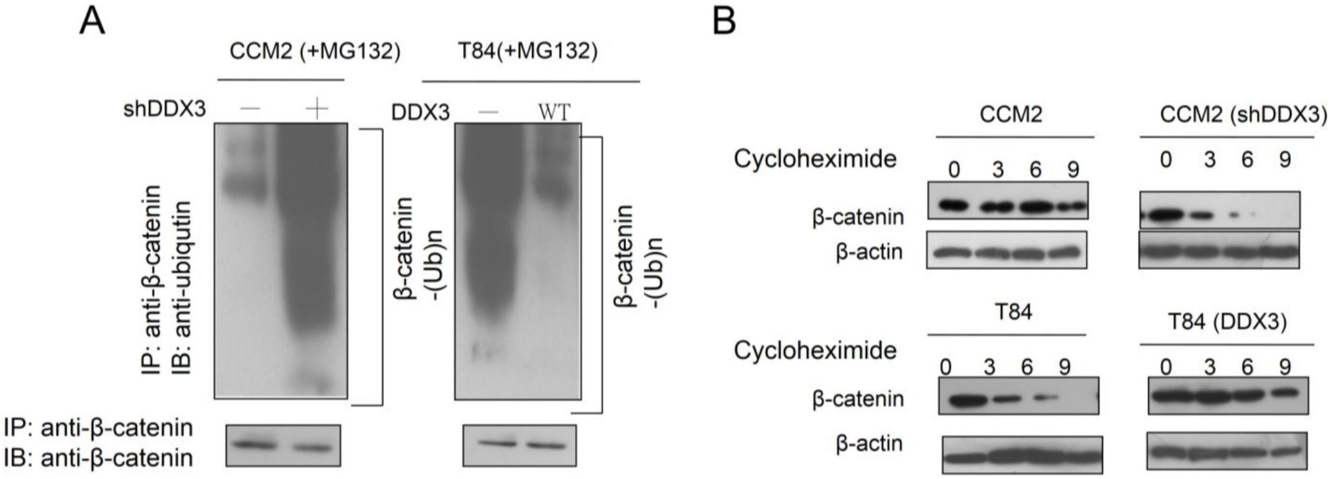

Supplementary Figure S2: DDX3 activates β-catenin/ TCF signaling via protecting β-catenin from ubiquitin-proteasome proteolysis. (**A**) CCM2 and T84 cells were transfected with DDX3 shRNA and its expression vector for 36 h. These cells were treated with 5 μmol/L MG132 for additional 5 hr and then cell lysates were immunoprecipitated with anti-β-catenin–conjugated beads and the immunoprecipitates were analyzed by SDS-PAGE, followed by immunoblotting with anti-ubiquitin antibody. (**B**) CCM2 cells were transfected with or without DDX3 shRNA and then co-transfected with Flag-β-catenin for 24 hr. T84 cells were transfected with DDX3 expression vector and then co-transfected with β-catenin expression vector for 24 hr. Both cells were incubated with cycloheximide (20 μg/ml) at the indicated time and then the cells were lysed to determine β-catenin levels using an anti-Flag antibody.

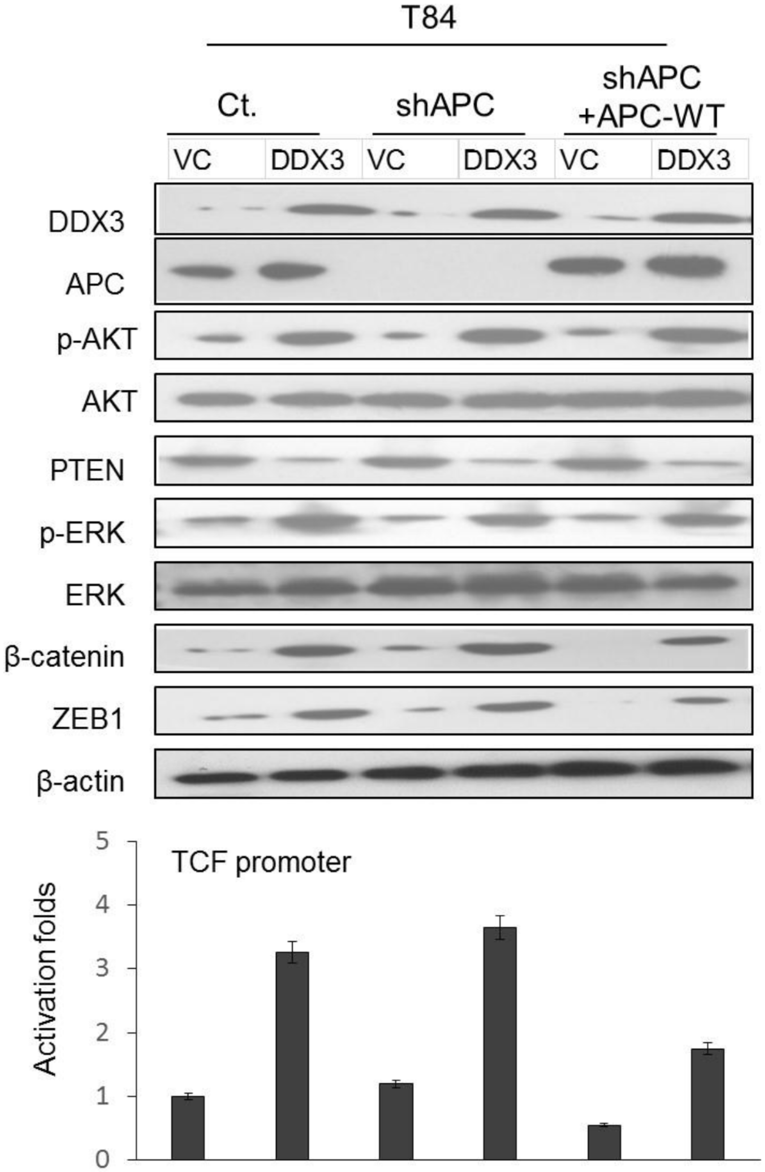

Supplementary Figure S3: DDX3 promoted cell invasion via activation of the β-catenin/ZEB axis in colorectal cancer regardless of APC mutations. T84 cells were transfected with indicated combination of DDX3, APC expression vector and APC (3′-UTR) shRNA for 48 hr. The expression of DDX3, KRAS, p-ERK, p-AKT, ERK, AKT and PTEN was determined by Western blotting.

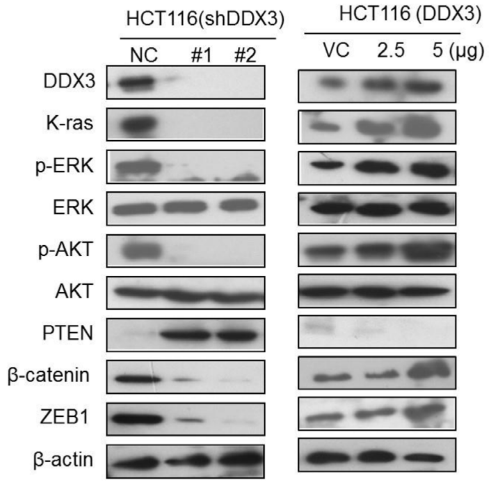

Supplementary Figure S4: DDX3 activate the β-catenin/ZEB1 axis in HCT116 cells which harbored β-catenin mutation and wild-type APC. Two kinds of shDDX3 and two doses of DDX3 expression vector were transfected into HCT116 cell lines. After 48 hr, the lysates were harvested and evaluated for levels of DDX3 KRAS, p-ERK, p-AKT, ERK, AKT, PTEN, β-catenin, ZEB1, and β-actin protein by Western blotting.

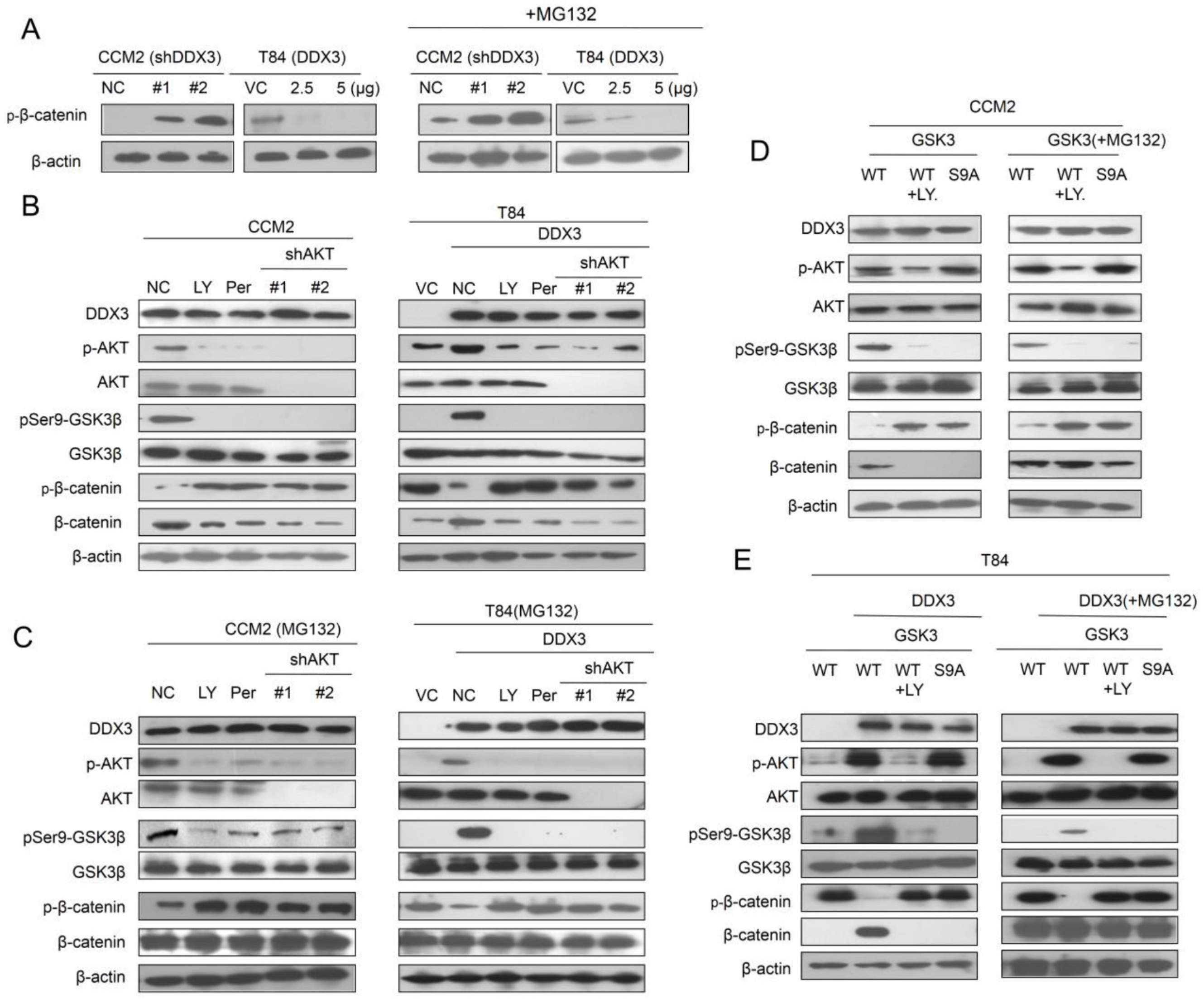

Supplementary Figure S5: DDX3 promotes β-catenin activation via decreased p-β-catenin expression due to suppressing GSK3β activity by Ser9 phosphorylation. (**A**) Western blotting was used to determine p-β-catenin (Serine-33, Serine-37 and threonine-41) levels in DDX3-konckdown CCM2 cells and DDX3-overexpressing T84 cells in the presence or absence of MG132 treatment for addition 5 hr. (**B, C**) CCM2 and DDX3-overexpressing T84 cells were treated with AKT inhibitors (LY294002, LY; Perifosine, Per) for 5 hr or two kinds of AKT shRNA for 36 hr in the presence or absence of MG132 for addition 5 hr. The cell lysates were used for SDS-PAGE, followed by immunoblotting with specific antibody. (**D**) CCM2 and (**E**) DDX3-overexpressing T84 cells were transfected with wild type (WT) or mutant (S9A) GSK3β and AKT inhibitor LY for 5 hr in the presence of absence of MG132 treatment. The cell lysates were used for SDS-PAGE, followed by immunoblotting with specific antibody.

Supplementary Table S1: KRAS, APC, β-catenin, and p53 mutation profile of human colorectal carcinoma cell lines

	**CCM2**	**CCM3**	**SW620**	**HCT15**	**T84**	**SW480**	**DLD1**
KRAS	G12V	G12V	G12V	G13D	G13D	G12V	G13D
APC	Q1338X (STOP codon)	Q1338X	Q1338X	R2166X; I1417fsX2	L1488fs*19	Q1338X	R2166X; I1417fsX2
β-catenin	WT	WT	WT	WT	WT	WT	WT
p53	R273H	R273H	R273H; P309S	S241F; C1101 – 2A > C	P60L; C376 – 1G > 2	R273H; P309S	S241F; C1101 – 2A > C
